# Improving extracellular production of *Serratia marcescens* lytic polysaccharide monooxygenase CBP21 and *Aeromonas veronii* B565 chitinase Chi92 in *Escherichia coli* and their synergism

**DOI:** 10.1186/s13568-017-0470-6

**Published:** 2017-09-07

**Authors:** Yalin Yang, Juan Li, Xuewei Liu, Xingliang Pan, Junxiu Hou, Chao Ran, Zhigang Zhou

**Affiliations:** Key Laboratory for Feed Biotechnology of the Ministry of Agriculture, Feed Research Institute, Chinese Academy of Agricultural Sciences, Beijing, 100081 People’s Republic of China

**Keywords:** Extracellular, Lytic polysaccharide monooxygenase, Chitinase, *Escherichia coli*, Synergism

## Abstract

Lytic polysaccharide monooxygenases (LPMOs) can oxidize recalcitrant polysaccharides and boost the conversion of the second most abundant polysaccharide chitin by chitinase. In this study, we aimed to achieve the efficient extracellular production of *Serratia marcescens* LPMO CBP21 and *Aeromonas veronii* B565 chitinase Chi92 by *Escherichia coli*. Twelve signal peptides reported with high secretion efficiency were screened to assess the extracellular production efficiency of CBP21 and Chi92, with glycine used as a medium supplement. The results showed that PelB was the most productive signal peptide for the extracellular production of CBP21 and Chi92 in *E. coli*. Furthermore, CBP21 facilitated the degradation of the three chitin substrates (colloidal chitin, β-chitin, and α-chitin) by Chi92. This study will be valuable for the industrial production and application of the two enzymes for chitin degradation.

**Electronic supplementary material:**

The online version of this article (doi:10.1186/s13568-017-0470-6) contains supplementary material, which is available to authorized users.

## Introduction

Chitin, a water insoluble poly-β-1,4-*N*-acetylglucosamine, is the second most abundant natural polysaccharide and is widely distributed in organisms such as fungi, arthropods and nematodes. Chitooligosaccharides (COS), the degraded products of chitin, have attracted increasing interest because of their physicochemical properties and potential food and pharmaceutical applications (Zou et al. 2016).

Efficient enzymatic conversion of recalcitrant chitin polymers is crucial for an economically and environmentally sustainable bioeconomy. However, the efficiency has been not desirable. The recently discovered lytic polysaccharide monooxygenases (LPMOs) are capable of oxidizing recalcitrant polysaccharides at different carbon positions [e.g., C1 (Vaaje-Kolstad et al. 2010), C4 (Isaksen et al. 2014)] and thus render the substrate more susceptible to hydrolysis by conventional glycoside hydrolases, providing new avenues toward more efficient enzymatic conversion of biomass. CBP21 is a chitin-active LPMO [Auxiliary activity family 10 (AA10)] from *Serratia marcescens* that can boost chitin depolymerization by *S. marcescens* chitinases (Suzuki et al. 1998; Vaaje-Kolstad et al. b, 2010). Also, CBP21 can promote the activity ChiA and ChiB from *Listeria monocytogenes* more efficiently than LmLPMO10 (Paspaliari et al. 2015). Moreover, CBP21 can enhance the activity of ChiC from *Aeromonas veronii* B565 by 2 (shrimp shell chitin as substrate) and 9 (colloidal chitin as substrate) folds (Huo et al. 2014). CBP21 is of economical importance for the environment-friendly chitin degradation.

Chitinase is a key enzyme in chitin degradation and production of soluble COS. *A. veronii* B565 isolated from aquaculture pond sediment was reported to be capable of producing chitinases that can be used to control Myxozoa-related or fungal diseases in fish (Li et al. 2011; Liu et al. 2011). Strain B565 encodes three chitinases from GH18, namely Chi92 (GenBank number AEB48885.1), ChiA (AEB48887.1), ChiB565 (AEB48892.1), two chitinases from GH19, namely ChiC (AEB50059.1) and ChiP (AEB48889.1). Among the five chitinases from *A. veronii* B565, Chi92 had high exochitinase specific activity on colloidal chitin and showed good potential as aquafeed additive enzyme (Huo et al. 2016; Zhang et al. 2014).

For industrial use, it is important that the costs associated with production of chitinolytic enzymes are low. Because of the advantages of rapid growth, high-yield and easy-to-scale production (Rosano and Ceccarelli 2014), *Escherichia coli* is the pre-eminent host for protein production both for commercial application [30% of recombinant therapeutic proteins, (Ferrer-Miralles et al. 2009)] and for research purposes [>70% of proteins, (Bill 2014)]. Extracellular protein production in *E. coli* is highly desirable as it simplifies downstream purification processes and increases product yield (Choi and Lee 2004). For *E. coli*, secreting recombinant protein extracellularly requires efficient crossing of two membrane barriers to overcome its low secretion capacity. Four strategies have been used for enhancing the secretory production of target proteins, i.e., selection of the signal peptide, coexpression of proteins to assist translocation and folding, improvement of periplasmic release, and protection of target proteins from degradation and contamination (Yoon et al. 2010). The combination of selection of the signal peptide and improvement of periplasmic release is the preferred strategy for efficient protein secretion. Signal peptides generally mediate transport of a protein from intracellular to the periplasmic space by either Sec or Tat pathway in *E. coli*. Owing to discrepancies between proteins, it is difficult to predict protein secretion efficiency. Selecting a suitable signal peptide is important for improving protein secretion. Moreover, the permeability enhancer glycine can replace the alanine residues in the peptide component of the cell wall peptidoglycan, thus enhancing the outer membrane permeability, and is frequently used to improve protein secretion (Hammes et al. 1973; Ding et al. 2010; Zou et al. 2014).

The extracellular production of CBP21 and Chi92 in *E. coli* has not been reported. In this study, we screened 12 signal peptides for efficient secretion of CBP21 and Chi92, all of which have been found effective for high protein secretion in *E. coli*, *Bacillus subtilis*, *Streptomyces lividans* or *Trichoderma reesei*. Moreover, glycine was chosen as a medium supplement to improve the production of extracellular CBP21 or Chi92. For improvement of the catalytic efficiency of Chi92, the synergistic effect of CBP21 on the chitin degradation rate of Chi92 was investigated.

## Materials and methods

### Strains, plasmids and growth conditions

All strains and plasmids were shown in Additional file 1: Table S1. *E. coli* DH5α (Transgen, Beijing, China) was used for gene cloning procedures. *E. coli* BL21 (DE3) (Novagen, Beijing, China) was used for recombinant protein production. *S. marcescens* GIM1.217 was purchased from Guangdong Microbiology Culture Center (GDMCC) and *A. veronii* B565 (CGMCC 3169) was a strain stored in our laboratory. Vector pET-28a(+) was used for cloning and expression of Chi92 and CBP21 genes. Cells were incubated at 37 °C in Luria broth with kanamycin (50 µg/mL).

### Plasmid construction

DNA polymerase LA Taq and T4 DNA ligase were obtained from Takara (Beijing, China), and restriction enzymes were obtained from New England Biolabs or Fermentas (Beijing, China). TIANpure Mini Plasmid and Gel purification kits were from Tiangen (Beijing, China). The used primers were summarized in Additional file 1: Table S2.

All genes encoding the signal peptides listed in Table 1 were synthesized by overlap PCR, which contain restriction sites for *Xba*I and *Eco*RI respectively. The synthesized DNA fragments were inserted into the *Xba*I and *Eco*RI sites of pET-28(a) to generate vectors containing different signal peptide: pET28a-SacB, pET28a-PelB, pET28a-TorA, pET28a-WompA, pET28a-OmpASIL2, pET28a-LMSEA, pET28a-LSEAmut, pET28a-Exyl, pET28a-gIII, pET28a-STII, pET28a-XCs or pET28a-CBHI, respectively.

**Table 1:** Signal peptides used in this study

Name	Amino acids sequence	Origin	References
WOmpA	MKKTAIAIAVALAGFATVAQA↓APKD	*E. coli*	(Robbens et al. 2006)
OmpASIL2 (OmpAS)	MKKTAIAIAVALAGFATVAQA↓SAPT	*E. coli*	(Robbens et al. 2006)
LM-SEA	MKKTAFTLLLFIALTLTTSPLVNG	*E. coli*	(Manuvera et al. 2010)
LSEA-mut (LSEAM)	MKKTAFTLLLFIALTWTTSPLASA	*E. coli*	(Manuvera et al. 2010)
StII	MKKNIAFLLASMFVFSIATNAYA	*E. coli*	(Chang et al. 1987)
PelB	MKYLLPTAAAGLLLLAAQPAMA	*Erwinia carotovora*	(Khushoo et al. 2005)
gIII	MKKLLFAIPLVVPFYSHS		(Lee et al. 2001)
Endoxylanase signal peptide (Exyl)	MFKFKKKFLVGLTAAFMSISMFSATASA	*Bacillus sp.*	(Choi et al. 2000)
SacB	MNIKKFAKQATVLTFTTALLAGGATQAFA	*B. subtilis*	(Wang et al. 2014)
CBHI	MYRKLAVISAFLATARAQS	*T. reesei*	(Zhong et al. 2011)
TorA	MNNNDLFQASRRRFLAQLGGLTVAGMLGPSLLTPRRATAAQAATDA	*E. coli*	(Yang et al. 2009)
Xylanase C signal peptide (XCs)	MQQDGTQQDRIKQSPAPLNGMSRRGFLGGAGTLALATASGLLLPGTAHA	*S. lividans*	(Miyazaki et al. 2013)

*Chi92* gene encoding chitinase Chi92 and *CBP21* gene encoding Lytic polysaccharide monooxygenase CBP21 (GenBank number MF150504) were amplified from the genomic DNAs of *S. marcescens* GIM1.217 and *A. veronii* strain B565, respectively. The amplified fragments without their respective signal peptide were cleaved by *Nco*I and *Xho*I and ligated into the above vectors containing different signal peptide to construct the plasmids as listed in Additional file 1: Table S1. The amplified fragments with or without their native signal peptide were cleaved by *Xba*I and *Xho*I and ligated into pET-28a(+) to construct the control plasmids as listed in Additional file 1: Table S1.

All plasmids were verified by DNA sequencing, and then the confirmed plasmids were separately transformed into competent *E. coli* DH5α or BL21 (DE3) cells.

### Protein production and purification

For production of each recombinant protein, first, *E. coli* BL21 (DE3) samples transformed with expression plasmids were cultured in Luria–Bertani broth with shaking at 200 rpm over night at 37 °C for use as a seed culture. Each culture was then diluted 1:100 with Terrific Broth medium with 50 μg/ml kanamycin (pH 6.4) and cultured at 37 °C, 200 rpm until the OD_600_ of bacteria culture was 0.8. Then lactose was added to a final concentration of 2% (w/v) to induce target protein production. To study whether the addition of glycine affects the excretion of Chi92 and CBP21, 1% (w/v) glycine was further supplemented after 12 h lactose-induction for another 12 h incubation. The culture supernatant and cell pellet were separated by centrifugation (4 °C, 10,000*g*, 10 min). The pellet was resuspended in PBS buffer to an OD_600_ of 10. Proteins in the supernatants were precipitated by adding 2 volumes of ice-cold acetone for 1 h at −20 °C and then centrifuged at 12,000*g* for 10 min at 4 °C. To ensure comparability between culture supernatant fraction and cell pellet fraction, the pellet from cell supernatant fraction (medium fraction, M) were resuspended in the same volume of PBS buffer as that used for cell pellet (cellular fraction, C). Fractions added with SDS-PAGE loading buffer were boiled at 100 °C for 10 min, then centrifuged (4 °C, 12,000*g*, 10 min) before loading on gels (TGX Stain-Free™ FastCast™ Acrylamide Kit, 12%, Bio-Rad, Cat. No. 161-0185) for SDS-PAGE. The same volume of each supernatant was loaded per lane.

Recombinant cells were resuspended in BugBuster protein extraction reagent at room temperature for 1 h and centrifugated to remove cell debris. The supernatant was incubated with pre-equilibrated Ni^2+^–NTA His·Bind resin at 4 °C for 1 h, and washed sequentially with 5 column volumes of elution buffer containing 20, 50 and 500 mM imidazole. Purified protein were analyzed using 12% SDS-PAGE.

### Preparation of chitin substrates

α-Chitin from shrimp shells was purchased from Sigma-Aldrich (V900332). Preparation of colloidal chitin from shrimp shell chitin (Sigma V900332) was according to the method described by Zhang et al. (2014). To prepare β-chitin, squid pens from the common squid fishes were dried and ground into powder, then treated with 8% HCl solution at a 1:14 (w/v) solid-liquid ratio  for 30 min at 25 °C for decalcification. After filtration, the sample was washed to neutral, and then treated by 10% NaOH solution at a 1:10 (w/v) for 2 h at 100 °C for deproteinization, filtered, washed to neutral and dried, and got β-chitin.

### Synergistic effects on enzymatic hydrolysis of chitin

To determine the influence of CBP21 on Chi92 efficiency, 250 µl of different contents (0, 0.005, 0.05, 0.5, 5, 50, 500 nmol) of CBP21 and 250 µl of 1% substrate (α-chitin, β-chitin or colloidal chitin) in sodium phosphate buffer (50 mM, pH 7.0) were incubated for 2 h at 37 °C, then 250 μl of Chi92 (21 nmol) was added and these mixtures were incubated for another 1 h at 37 °C. At the end of reaction, the released reducing sugars were measured by the 3,5-dinitrosalicylic acid assay (Liu et al. 2011). All reactions were performed in triplicate.

## Results

### Signal peptide for the efficient extracellular production of CBP21 and Chi92 in *E. coli*

**Fig. 1:**
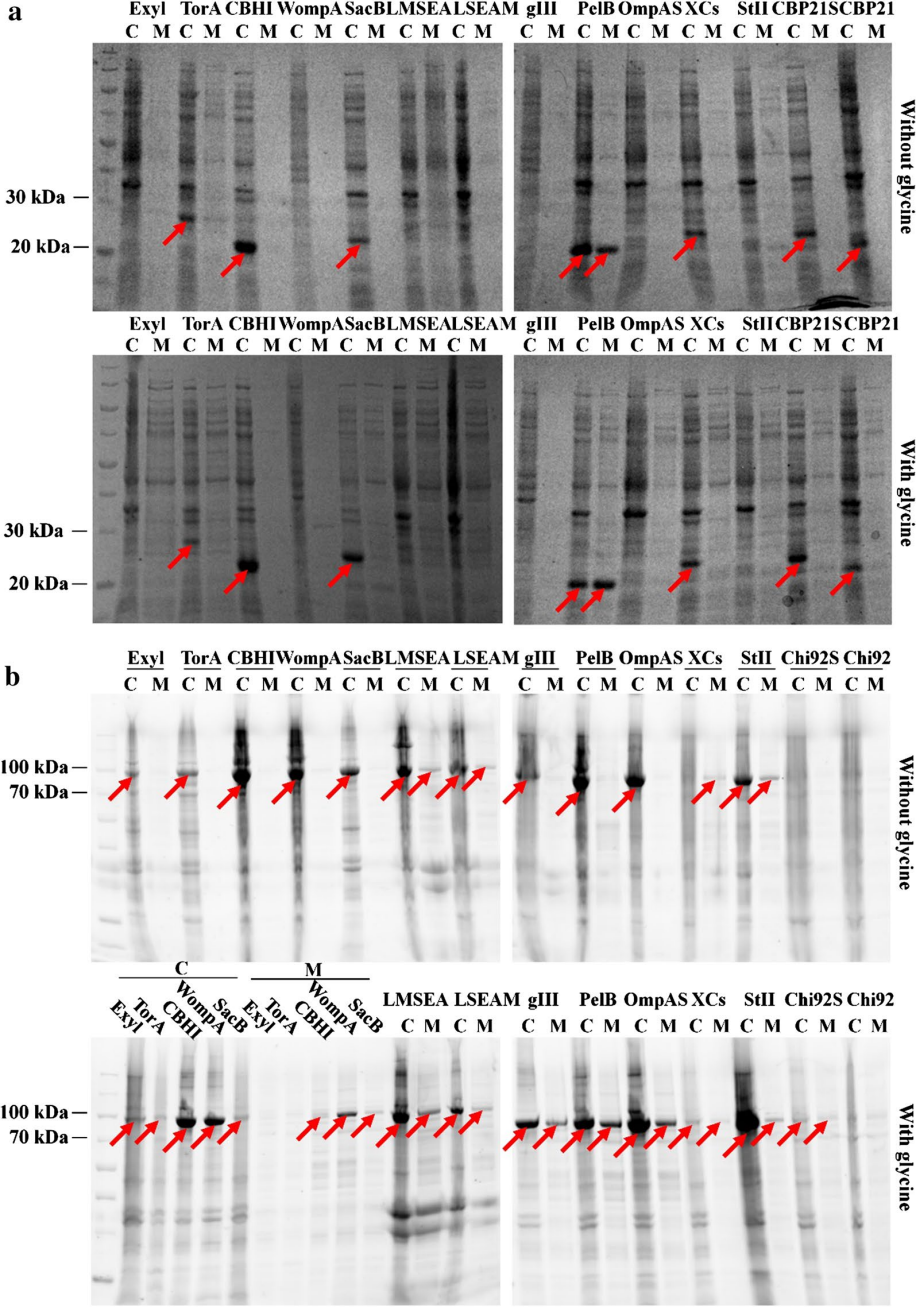
Comparison of the extracellular production of CBP21 or Chi92 fused with different signal peptides. Recombinant *E. coli* cells were constantly cultured for 24 h in TB medium with lactose-induction or in TB medium with 1% glycine addition after 12 h lactose-induction followed by another 12 h incubation. The intracellular (*C*) and extracellular (*M*) production of CBP21 (**a**) or Chi92 (**b**) fused with different signal peptides (as indicated in the *top of each lane*) were analysed by SDS-PAGE. CBP21S and Chi92S represent CBP21 and Chi92 with their native signal peptide, respectively. CBP21 and Chi92 represent CBP21 and Chi92 without any signal peptide, respectively. The *red arrows* indicate the CBP21s and Chi92s with molecular weight estimation of 20–25 or 91–96 kDa, respectively

Since signal peptides can differ greatly in their ability for the secretion of a given protein, a signal peptide screening procedure is crucial for efficient secretion. To select the suitable signal peptide for the efficient extracellular production of CBP21 or Chi92, 12 signal peptides were fused respectively to the N-terminus of CBP21 or Chi92, and the intracellular and extracellular production of CBP21 or Chi92 was analyzed by SDS-PAGE. Two signal peptides, CBHI and PelB, significantly increased the production level of CBP21 (Fig. 1a). In addition, PelB can efficiently increase the release of intracellular CBP21 to extracellular milieu of recombinant *E. coli*.

To enhance the extracellular production of CBP21, recombinant cells after 12 h lactose induction were further cultured with 1% glycine for 12 h. Compared with the absence of glycine, glycine supplementation increased the amount of extracellular total protein from recombinant *E. coli* with pPelB-CBP21 (from 0.83 to 0.94 mg/ml). Furthermore, the intensity [Area of 5188 using Iimage J analysis (NIH, USA, Version: ij150-win-jre6-32-bit)] of extracellular CBP21 band in glycine-supplementation group is higher than that (Area of 5675) in the non-glycine control group (Fig. 1a), indicating that glycine supplementation increased the extracellular production of PelB-fused CBP21 from recombinant *E. coli*.

As shown in Fig. 1b, most signal peptides, such as CBHI, WompA, LMSEA, LSEA-mut, gIII, PelB, OmpASIL2 and StII, significantly increased the production level of Chi92. Furthermore, signal peptides StII, LMSEA, LSEA-mut and XCs can secrete Chi92 to the extracellular medium of recombinant *E. coli*. Compared with the respective controls, several signal peptides, such as PelB, OmpASIL2, WompA, LMSEA and gIII, markedly improved the extracellular production level of Chi92 from recombinant *E. coli* cultured in glycine supplemented medium. Collectively, PelB was the most productive signal peptide for the extracellular production of CBP21 and Chi92 in *E. coli* cultured in glycine supplemented medium.

### Enhancement of the hydrolytic activity of Chi92 on various chitin substrates by CBP21

**Fig. 2:**
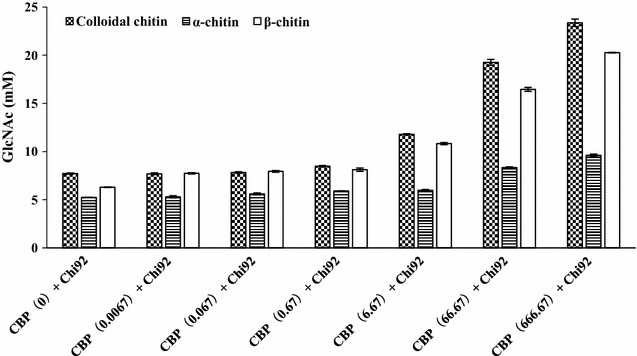
Dose-response effects of CBP21 on the activity of Chi92 to colloidal chitin, α-chitin or β-chitin. 250 µl of different contents (0, 0.005, 0.05, 0.5, 5, 50, 500 nmol) of CBP21 and 250 µl of 1% substrate (colloidal chitin, α-chitin or β-chitin) in sodium phosphate buffer (50 mM, pH 7.0) were incubated in 37 °C for 2 h, then 250 μl of Chi92 (21 nmol) was added and these reaction mixtures were incubated in 37 °C for another 1 h. Sample were measured by the 3,5-dinitrosalicylic acid (DNS) assay. Each data value represents the average of three measurements

Different concentrations of CBP21 were first pre-incubated with the three substrates for 2 h, then purified Chi92 was added to hydrolyze substrates for 1 h. The results showed a dose-dependent synergistic effect of CBP21 on the hydrolytic efficiency of Chi92. Chi92 displays maximum degradation rates at the CBP21 concentration of 666.67 µM. In the presence of 66.67 or 666.67 µM CBP21, Chi92 incubated with colloidal chitin, α-chitin and β-chitin produced 2.49 or 3.03-times, 1.59 or 1.84-times and 2.61 or 3.22-times of the product of the control group, respectively (Fig. 2). These results demonstrated that CBP21 facilitated the degradation of three chitin substrates by Chi92.

## Discussion

Signal peptides showed large variations in secretion efficiencies, depending on the nature of the secreted protein. A suitable signal peptide for one protein may not be efficient for another protein (Low et al. 2013). A similar outcome was obtained in the present study, all 12 selected signal peptides increased the production level of Chi92 in varying degrees, whereas only four signal peptides, including PelB, CBHI, SacB and XCs, increased the production level of CBP21. Without glycine addition, StII, LMSEA, LSEA-mut and XCs can efficiently improve the release of intracellular Chi92 to culture media, while only PelB can promote the release of CBP21 to culture media with high efficiency. The cleavage efficiency of the signal peptide affects protein secretion levels (Low et al. 2013). Interleukin-2 production in *E. coli* periplasm was increased drastically by a single amino acid substitution in OmpA cleavage site (Robbens et al. 2006). Substitution of the *Staphylococcus staphilococcic* enterotoxin A leader sequence of Val–Asn–Gly with a conserved *E. coli* signal peptidase recognition sequence of Ala–Ser–Ala significantly increased enterotoxin A secretion to the periplasm (Manuvera et al. 2010). The modified signal peptide (OmpASIL2 and LSEA-mut) and their respective controls (WompA and LMSEA) reported previously (Manuvera et al. 2010; Robbens et al. 2006) were also used for the secretion of CBP21 or Chi92, but the reported improvements in efficiency were not observed in CBP21 or Chi92, which is consistent with the variation in secretion efficiency of signal peptides for different proteins. The effects of glycine on the extracellular production of various signal peptides are also different. Glycine addition can significantly improve the extracellular production of Chi92 fused with PelB, WOmpA, OmpASIL2, gIII or LMSEA, but can’t improve the extracellular production of StII- and CBHI-fused Chi92. This may be due to the differences in the ability of the different signal peptides to transport protein to periplasm.

CBP21 binds stronger to β-chitin than to α-chitin (Suzuki et al. 1998; Vaaje-Kolstad et al. b). As shown in Table 2, most of the reported chitin-active LPMOs tend to bind β-chitin, and some chitin-active LPMOs bind equally well to α-chitin and β-chitin, and a few chitin-active LPMOs strictly bind to α-chitin. Substrate accessibility should be a key determinant for binding preference of LPMOs. The interaction of the chitin chains in β-chitin is more loosely than in α-chitin, and β-chitin has a higher water content and easier accessibility (Rinaudo 2006; Vaaje-Kolstad et al. 2005a), which could explain why most LPMOs showed higher affinity for β-chitin than for α-chitin. The conserved exposed aromatic residue in the substrate-binding surface of LPMOs seems to have impact on binding affinity. CBP21, with higher binding preference to β-chitin, has a Tyr in this position (Tyr54) (Suzuki et al. 1998; Vaaje-Kolstad et al. b), whereas CHB1 and CHB2 from *Streptomyces avermitilis* and *Streptomyces reticuli*, which specifically binding α-chitin, have a Trp in this position (W57 and W56, respectively) (Kolbe et al. 1998; Zeltins and Schrempf 1997). Mutation of the corresponding residue in LPMOs impacted either substrate-binding preferences or synergism (Nakagawa et al. 2015; Vaaje-Kolstad et al. b; Zeltins and Schrempf 1997).

**Table 2:** Binding preferences of chitin-active LPMOs

Name	Source	Binding perference	References
CBP21	*S. marcescens*	β-chitin > colloidal chitin > α-chitin	(Suzuki et al. 1998; Vaaje-Kolstad et al. b)
ChbB	*Bacillus amyloliquefaciens*	β-chitin > α-chitin	(Chu et al. 2001)
E7	*Thermobifida fusca*	β-chitin > α-chitin > microcrystalline cellulose	(Moser et al. 2008)
SpCBP21	*Serratia proteamaculans*	β-chitin > α-chitin	(Purushotham et al. 2012)
SpCBP50	*S. proteamaculans*	β-chitin > α-chitin	(Purushotham et al. 2012)
BtCBP	*Bacillus thuringiensis*	β-chitin > α-chitin	(Manjeet et al. 2013)
BliCBP	*B. licheniformis*	β-chitin > α-chitin	(Manjeet et al. 2013)
SgLPMO10F	*Streptomyces griseus*	β-chitin > α-chitin	(Nakagawa et al. 2015)
JdLPMO10A	*Jonesia denitrificans*	β-chitin > α-chitin	(Mekasha et al. 2016)
LmLPMO10	*Listeria monocytogenes*	β-chitin slightly > α-chitin	(Paspaliari et al. 2015)
EfCBM33A	*Enterococcus faecalis*	β-chitin slightly > α-chitin	(Vaaje-Kolstad et al. 2012)
LlCBP33A	*Lactococcus lactis*	α-chitin ≈ β-chitin > Avicel > colloidal chitin > chitin beads	(Vaaje-Kolstad et al. 2009)
BtLPMO10A	*B. thuringiensis*	α-chitin ≈ β-chitin > chitin beads > colloidal chitin	(Zhang et al. 2015)
*Cj*LPMO10A	*Cellvibrio japonicus*	α-chitin > β-chitin	(Forsberg et al. 2016)
CHB1	*Streptomyces olivaceoviridis*	Strictly to α-chitin	(Zeltins and Schrempf 1997)
CHB2	*Streptomyces reticuli*	Strictly to α-chitin	(Kolbe et al. 1998)
CHB3	*Streptomyces coelicolor*	α-chitin > β-chitin	(Saito et al. 2001)

**Fig. 3:**
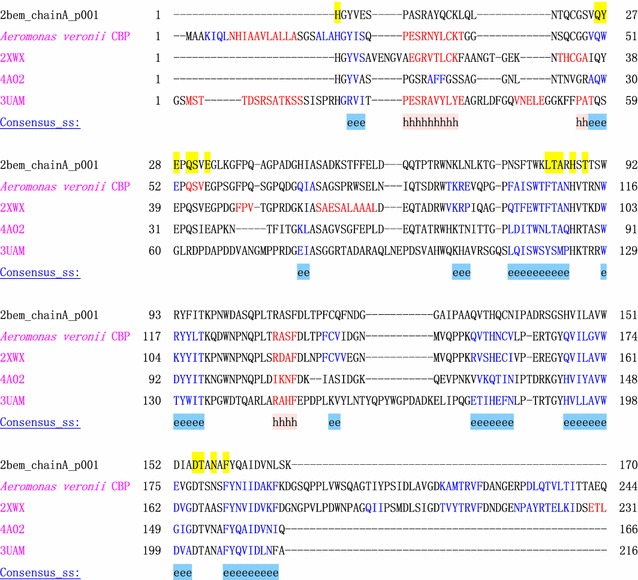
Alignment of CBP21 from *S. marcescens* (PDB ID: 2BEM), GbpA from *Vibrio cholerae* (PDB ID: 2XWX) (Wong et al. 2012), Efcbm33a from *Enterococcus faecalis* (PDB ID: 4A02) (Vaaje-Kolstad et al. 2012), Chitin Binding Domain from *Burkholderia pseudomallei* (PDB ID: 3UAM) and CBP from *A. veronii* B565 using PROMALS3D (http://prodata.swmed.edu/promals3d/promals3d.php). Predicted secondary structures: red h: alpha-helix; blue e: beta-strand. Residues involved in chitin binding based on a 2H/1H exchange experiment (Q53, Q57, S58, L110, T111, A112, H114 and T116 of CBP21) (Aachmann et al. 2012) or determined by site-directed mutagenesis (Y54, E55, E60, H114, D182 and N185 of CBP21) (Vaaje-Kolstad et al. b) or highly conserved residues on the CBP21 substrate binding surface determined by ConSurf analysis (H28, E55, S58, E60, T111, A112, H114, D182, T183, N185 and F186 of CBP21) (Ashkenazy et al. 2010) showed by yellow surface coloring

**Fig. 4:**
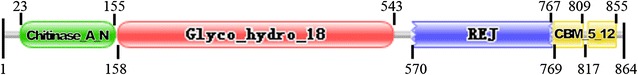
Schematic representation of domains architecture of Chi92 from *A. veronii* B565. ChitinaseA_N: Chitinase A, N-terminal domain; Glyco_hydro_18: Glycosyl hydrolases family 18; REJ: REJ (Receptor for Egg Jelly) domain; CBM_5_12: Carbohydrate binding domain

Different LPMOs had different synergy effects on a given chitinase. CBP21 was able to boost the activity of both ChiA and ChiB from *Listeria monocytogenes* more than LmLPMO10 (Paspaliari et al. 2015). *A. veronii* B565 also encodes a chitin binding protein (CBP) (AEB51592.1) [Auxiliary activity family 10 (AA10)]. Our unpublished research found that CBP had no effect on the activity of Chi92 to colloidal chitin or α-chitin compared with CBP21 (data not shown). CBP contains 9 of the 11 key amino acid residues present on the substrate binding surface of CBP21 (Fig. 3). The other two key residues Y54 and L110 in CBP21, which were associated with chitin binding on the CBP21 surface, were replaced by W51 and F107 in the corresponding positions of CBP (Fig. 3). Whether the two residues affect the auxiliary activity of CBP to Chi92 requires further investigation. CBP21 had differential effect on the activity of different chitinases. CBP21 greatly boosted the activities of exo-acting GH18s ChiA and ChiB (Hamre et al. 2015). Oxidative cleavage of recalcitrant polysaccharide glycosidic bonds by a LPMO has been presumed to generate new chain ends as new attacking points for chitinases or cellulases (Vaaje-Kolstad et al. 2010). Like ChiA and ChiB, Chi92 is an exo-acting GH18 (Fig. 4) (Huo et al. 2016), and the synergistic effect of Chi92 with CBP21 also suggested that new ends generated by CBP21 are key for the enhancement of Chi92 activity. However, CBP21 had no effect on the initial rate of the endo-acting GH18 ChiC (Hamre et al. 2015). The mechanism underlying the specificity of synergy between LMPO and chitinase deserves further study.

## Additional file

**Additional file 1.** Additional tables.

## Abbreviations

LPMOs: lytic polysaccharide monooxygenases
CBP: chitin binding protein
AA10: auxiliary activity family 10
COS: chitooligosaccharides
GH18: glycoside hydrolase family 18
GH19: glycoside hydrolase family 19
