# Supplementary material for: Improving extracellular production of Serratia marcescens lytic polysaccharide monooxygenase CBP21 and Aeromonas veronii B565 chitinase Chi92 in Escherichia coli and their synergism
Source: AMB Express. 2017 Sep 7;7:170. doi: 10.1186/s13568-017-0470-6 (PMC5589716; doi:10.1186/s13568-017-0470-6)
Supplement: Supplementary file 1 — Additional file 1. Additional tables. [file 13568_2017_470_MOESM1_ESM.docx]

**Additional Information**

**Additional Tables**

**Table S1. Bacterial strains and plasmids used in this study**

| **Strain/Plasmid** | **Description** | **Source** |
| --- | --- | --- |
| *E. coli* DH5α | F^-^ φ80 *lac*Z△M15 △(lacZYA-argF) U169 *end*A1 *rec*A1 *hsd*R17(r_k_^-^, m_k_^+^) *sup*E44 λ^-^ thi-1 *gyr*A96 *rel*A1 *pho*A | Tiangen |
| E. coli BL21 (DE3) | F ^–^ *ompT hsdS_B_* (*r_B_^–^ m_B_^–^*) *gal dcm* (DE3) | Novagen |
| *Serratia marcescens* GIM1.217 | A strain produces chitin-degrading enzyme. | GDMCC |
| *Aeromonas veronii* strain B565 (CGMCC 3169) | A gram-negative, rod-shaped bacterium, with the ability to degrade chitin, was isolated from aquaculture pond sediment in China. | (Li et al. 2011) |
| pET28a | 5.4 kb, f1 ori, T7 promoter, Kan^R^ | Novagen |
| pET22b | 5.4 kb, f1 ori, T7 promoter, Amp^R^ | Novagen |
| pET28a-PelB | a signal peptide (SP) PelB gene in *Xba*I-*Eco*RI site of pET28a | This study |
| pET28a-SacB | a SP SacB gene in *Xba*I-*Eco*RI site of pET28a |  |
| pET28a-TorA | a SP TorA gene in *Xba*I-*Eco*RI site of pET28a | This study |
| pET28a-WompA | a SP WompA gene in *Xba*I-*Eco*RI site of pET28a | This study |
| pET28a-OmpASIL2 | a SP OmpASIL2 gene in *Xba*I-*Eco*RI site of pET28a | This study |
| pET28a-LMSEA | a SP LMSEA gene in *Xba*I-*Eco*RI site of pET28a | This study |
| pET28a-LSEAmut | a SP LSEAmut gene in *Xba*I-*Eco*RI site of pET28a | This study |
| pET28a-Exyl | a SP Exyl gene in *Xba*I-*Eco*RI site of pET28a | This study |
| pET28a-gIII | a SP gIII gene in *Nde*I-*Nco*I site of pET28a-PelB | This study |
| pET28a-STII | a SP STII gene in *Xba*I-*Eco*RI site of pET28a | This study |
| pET28a-XCs | a SP XCs gene in *Xba*I-*Eco*RI site of pET28a | This study |
| pET28a-CBHI | a SP CBHI gene in *Xba*I-*Eco*RI site of pET28a | This study |
| pSacB-CBP21 | a CBP21 gene without SP sequence in *Nco*I*-Xho*I site of pET28a-SacB | This study |
| pPelB-CBP21 | a CBP21 gene without SP sequence in *Nco*I*-Xho*I site of pET28a-PelB | This study |
| pTorA-CBP21 | a CBP21 gene without SP sequence in *Nco*I*-Xho*I site of pET28a-TorA | This study |
| pWompA-CBP21 | a CBP21 gene without SP sequence in *Nco*I*-Xho*I site of pET28a-WompA | This study |
| pOmpASIL2 -CBP21 | a CBP21 gene without SP sequence in *Nco*I*-Xho*I site of pET28a- OmpASIL2 | This study |
| pLMSEA-CBP21 | a CBP21 gene without SP sequence in *Nco*I*-Xho*I site of pET28a-LMSEA | This study |
| pLSEAmut-CBP21 | a CBP21 gene without SP sequence in *Nco*I*-Xho*I site of pET28a-LSEAmut | This study |
| pExyl-CBP21 | a CBP21 gene without SP sequence in *Nco*I*-Xho*I site of pET28a-Exyl | This study |
| pgIII-CBP21 | a CBP21 gene without SP sequence in *Nco*I*-Xho*I site of pET28a-gIII | This study |
| pSTII-CBP21 | a CBP21 gene without SP sequence in *Nco*I*-Xho*I site of pET28a-STII | This study |
| pXCs-CBP21 | a CBP21 gene without SP sequence in *Nco*I*-Xho*I site of pET28a-XCs | This study |
| pCBHI-CBP21 | a CBP21 gene without SP sequence in *Nco*I*-Xho*I site of pET28a-CBHI | This study |
| pET-CBP21S | a CBP21 gene containing SP sequence in *Nco*I*-Xho*I site of pET28a | This study |
| pET-CBP21 | a CBP21 gene without SP sequence in *Nco*I*-Xho*I site of pET28a | This study |
| pSacB-Chi92 | a Chi92 gene without SP sequence in *Nco*I*-Xho*I site of pET28a-SacB | This study |
| pPelB-Chi92 | a Chi92 gene without SP sequence in *Nco*I*-Xho*I site of pET28a-PelB | This study |
| pTorA-Chi92 | a Chi92 gene without SP sequence in *Nco*I*-Xho*I site of pET28a-TorA | This study |
| pWompA-Chi92 | a Chi92 gene without SP sequence in *Nco*I*-Xho*I site of pET28a-WompA | This study |
| pOmpASIL2 -Chi92 | a Chi92 gene without SP sequence in *Nco*I*-Xho*I site of pET28a- OmpASIL2 | This study |
| pLMSEA-Chi92 | a Chi92 gene without SP sequence in *Nco*I*-Xho*I site of pET28a-LMSEA | This study |
| pLSEAmut-Chi92 | a Chi92 gene without SP sequence in *Nco*I*-Xho*I site of pET28a-LSEAmut | This study |
| pExyl-Chi92 | a Chi92 gene without SP sequence in *Nco*I*-Xho*I site of pET28a-Exyl | This study |
| pgIII-Chi92 | a Chi92 gene without SP sequence in *Nco*I*-Xho*I site of pET28a-gIII | This study |
| pSTII-Chi92 | a Chi92 gene without SP sequence in *Nco*I*-Xho*I site of pET28a-STII | This study |
| pXCs-Chi92 | a Chi92 gene without SP sequence in *Nco*I*-Xho*I site of pET28a-XCs | This study |
| pCBHI-Chi92 | a Chi92 gene without SP sequence in *Nco*I*-Xho*I site of pET28a-CBHI | This study |
| pET-Chi92S | a Chi92 gene containing SP sequence in *Nco*I*-Xho*I site of pET28a | This study |
| pET-Chi92 | a Chi92 gene without SP sequence in *Nco*I*-Xho*I site of pET28a | This study |

**Table S2. Primers used in this study**

| **Primers** | **Sequence** |
| --- | --- |
| SignalpeptideF | TCGCTCTAGAAATAATTTTGTTTAACTTTAAGA |
| SignalpeptideR | CGAATTCGGATCCGAATTAATTCCGATATCCATGGC |
| SignalpeptideF1 | TCCCCTCTAGAAATAATTTTGTTTAACTTTAAGAAGGAGATATACATATG |
| WOmpAR1 | GCCAGTGCCACTGCAATCGCGATAGCTGTCTTTTGCATATGTATATCTCCTTC |
| WOmpAF2 | GCGATTGCAGTGGCACTGGCTGGTTTCGCTACCGTAGCGCAG |
| WOmpAR2 | AATTCCGATATCCATGGCATCTTTCGGAGCGGCCTGCGCTACGGTAGCGAAAC |
| OmpASIL2R2 | AATTCCGATATCCATGGCGATGCGAGCTGCGGCCTGCGCTACGGTAGCGAAAC |
| LMSEAR1 | AAAAGTAATGTAAATGCTGTTTTTTTCATATGTATATCTCCTTC |
| LMSEAF2 | AGCATTTACATTACTTTTATTCATTGCCCTAACGTTGACAACAAGTCCACTTG |
| LMSEAR2 | AATTCCGATATCCATGGCACCATTTACAAGTGGACTTGTTGTC |
| LSEAmutR2 | AATTCCGATATCCATGGCCGACGCAAGTGGACTTGTTGTC |
| ExylR1 | AATCCCACTAAGAATTTCTTTTTAAACTTAAACATATGTATATCTCCTTC |
| ExylF2 | GAAATTCTTAGTGGGATTAACGGCAGCTTTCATGAGTATCAGCATGTTTTCG |
| ExylR2 | AATTCCGATATCCATGGCTGCAGAGGCGGTTGCCGAAAACATGCTGATACT |
| gIIIR1 | GGAACAACTAAAGGAATTGCGAACAGCAGTTTTTTCATATGTATATCTCCTTC |
| gIIIF2 | AATTCCTTTAGTTGTTCCTTTCTATTCTCACTCCGCCATG |
| gIIIR2 | TTAATTCCGATATCCATGGCGGAGTGAGAATAGAAA |
| gIIIF3 | TATACATATGAAAAAACTGCTGT |
| gIIIR3 | ATATCCATGGCGGAGTGAGAATAG |
| STIIR1 | TGCAAGAAGAAATGCGATATTCTTTTTCATATGTATATCTCCTTC |
| STIIF2 | ATCGCATTTCTTCTTGCATCTATGTTCGTTTTTTCTATTGCTACAAATG |
| STIIR2 | AATTCCGATATCCATGGCTGCATAGGCATTTGTAGCAATAGAA |
| XCsR1 | GACTCTGCTTGATCCGGTCCTGCTGTGTGCCGTCCTGCTGCATATGTATATCTCCTTC |
| XCsF2 | ACCGGATCAAGCAGAGTCCCGCCCCTCTCAACGGAATGA |
| XCsR2 | CGAGGGTGCCGGCGCCACCGAGGAAGCCTCTGCGGCTCATTCCGTTGAGAG |
| XCsF3 | GTGGCGCCGGCACCCTCGCGCTCGCTACCGCGTCCGGGC |
| XCsR3 | ATTCCGATATCCATGGCGGCGTGGGCTGTGCCGGGCAGCAGCAGCCCGGACGCGGTAGC |
| CBHIF | AAGAAGGAGATATACATATGTACCGTAAACTGGCTGTAATCTCTGCTTTTTTGGCTA |
| CBHIR | TTAATTCCGATATCCATGGCAGACTGAGCACGAGCGGTAGCCAAAAAAGCAGAGA |
| SacBF1 | AAGAAGGAGATATACATATGAACATCAAAAAGTTTGCA |
| SacBFR1 | TTAATTCCGATATCCATGGCCGCAAAAGCTTGAGTTG |
| TorAF | TATACATATGAACAATAACGATCTCTTTCAGGCAT |
| TorAR | TATCCATGGCTTGCGCCGCAGTCGCACGTCG |
| Chi92SF | TATACCATGGGCCTAAGTCCAAAGCCTACCAT |
| Chi92SR | GTGCTCGAGTTTACAACTGGCGGCTCCCACATCCTG |
| Chi92F | TATACCATGGGCgcggcgcccggcaagccgaccatag |
| CBP21SF | TATACCATGGGCAACAAAACTTCCCGTACCCTGC |
| CBP21SR | GTGGTGCTCGAGTTTACTCAGGTTGACGTCG |
| CBP21F | TATACCATGGGCCACGGTTATGTCGAATCG |

**References:**

Li Y, Liu Y, Zhou Z, Huang H, Ren Y, Zhang Y, Li G, Zhou Z, Wang L (2011) Complete genome sequence of *Aeromonas veronii* strain B565. J Bacteriol 193(13):3389-3390. doi:10.1128/JB.00347-11
